# Improved Safety, Bioavailability and Pharmacokinetics of Zidovudine through Lactoferrin Nanoparticles during Oral Administration in Rats

**DOI:** 10.1371/journal.pone.0140399

**Published:** 2015-10-13

**Authors:** Prashant Kumar, Yeruva Samrajya Lakshmi, Bhaskar C., Kishore Golla, Anand K. Kondapi

**Affiliations:** Department of Biotechnology and Bioinformatics, School of Life Sciences, University of Hyderabad, Hyderabad, 500046, India; University of Pittsburgh, UNITED STATES

## Abstract

Zidovudine (AZT) is one of the most referred antiretroviral drug. In spite of its higher bioavailability (50–75%) the most important reason of its cessation are bone marrow suppression, anemia, neutropenia and various organs related toxicities. This study aims at the improvement of oral delivery of AZT through its encapsulation in lactoferrin nanoparticles (AZT-lactonano). The nanoparticles (NPs) are of 50–60 nm in size and exhibit 67% encapsulation of the AZT. They are stable in simulated gastric and intestinal fluids. Anti-HIV-1 activity of AZT remains unaltered in nanoformulation in acute infection. The bioavailability and tissue distribution of AZT is higher in blood followed by liver and kidney. AZT-lactonano causes the improvement of pharmacokinetic profile as compared to soluble AZT; a more than 4 fold increase in AUC and AUMC in male and female rats. The serum C_max_ for AZT-lactonano was increased by 30%. Similarly there was nearly 2-fold increase in T_max_ and t_1/2_. Our in vitro study confirms that, the endosomal pH is ideal for drug release from NPs and shows constant release from up to 96h. Bone marrow micronucleus assay show that nanoformulation exhibits approximately 2fold lower toxicity than soluble form. Histopathological and biochemical analysis further confirms that less or no significant organ toxicities when nanoparticles were used. AZT-lactonano has shown its higher efficacy, low organs related toxicities, improved pharmacokinetics parameter while keeping the antiviral activity intact. Thus, the nanoformulation are safe for the target specific drug delivery.

## Introduction

Zidovudine is the first drug approved for the treatment of HIV infection and is still in the part of the first line regimen in Highly Active Antiretroviral Therapy (HAART) [1]. Despite of its efficacy, the factors that limit its clinical use are its toxicity, suboptimal bioavailability and pharmacokinetics which includes bone marrow aplasia [2], inhibition of mitochondrial machinery [3], short plasma half-life [4] and high hepatic first-pass metabolism [5] etc. This will eventually lead to the increase in the frequency and dosage of the regimen resulting in the unwanted side effects that compromise in the adherence to the antiretroviral treatment. AZT (300 mg twice a day oral 1 mg per kg intravenous infusion over 1 hour every 4 hours), was the first drug approved by the Food and Drug Administration (FDA). Commercially, AZT is available in various forms such as capsules, tablets, syrup and intravenous injection. Further, FDA has approved the fixed tablet formulation of AZT for HIV naive patient in combination with other ART drugs, these includes *Combivir* (300mg AZT plus 150mg lamivudine) and *Trizivir* (300mg AZT plus 150mg lamivudine plus 300mg abacavir) [6]. This necessitates zero order and targeted delivery of AZT since excess plasma concentration occur immediately after its administration [7]. Hence, a successful treatment of HIV infection requires a uniform systemic level of the drug throughout the course of the therapy.

Apart from T-lymphocytes, reticuloendothelial cells namely monocytes and macrophages act as major reservoirs for HIV and are thought to be responsible for its distribution throughout the body and brain [8–11]. So a sterilizing cure for HIV is not possible without the access of drug regimens into macrophage system. In addition, AZT is reported to be a substrate of diverse drug efflux mechanisms present in cells of CNS, Immune system and Intestinal epithelium which is mainly mediated by ATP binding cassette (ABC) family of proteins viz., P-glycoprotein [12]. This consequently will lead to the evolution of drug resistant strains. This is one of the main reasons of the intra- and inter-patient variability and nonlinearity observed in the bioavailability of AZT [13,14]. To circumvent all of the above limitations associated with AZT delivery various methods were employed that encompasses delivery as prodrugs, encapsulation in polymeric and non-polymeric nanocarriers like nanoconjugates, micelles, surface engineered liposomes, SLNs etc. [15–18]. The various advantages in using nanoparticles as delivery vehicles are their size, surface charge, large surface area to volume ratio, stability, multifunctional and biomimetic properties [19–21].

In spite of having so much of beneficial record of AZT [22], the previous (pharmacokinetics) PK studies showed that, AZT is responsible for the bone marrow toxicity [23] which leads to bone marrow suppression [24] and finally results to various alteration related to hematopoiesis [25–27]. Further AZT has been proven as a genotoxic compound [28,29] which cause the induction of micronuclei in the mouse bone marrow cells [30].

Earlier research in our lab involved in the development of nanoformulation of doxorubicin, carboplatin and curcumin with transferrin family of proteins namely apotransferrin and lactoferrin and these were successfully applied for the treatment of hepatocellular carcinoma in rats and HIV-1 infection in cell line [31–35]. Since oral administration is the best modality available for the drug delivery, the present work involves the improvement in the delivery of AZT using the lactoferrin as nanocarrier system. So the advantage with the present nanoformulation of AZT with lactoferrin is bidirectional, where the carrier itself has antiviral activity along with AZT itself. The present work showed the improved safety, bioavailability and pharmacokinetics of AZT encapsulated in lactoferrin nanoparticles (AZT-lactonano).

## Materials and Methods

### Materials

Lactoferrin and olive oil used for preparation of nanoparticle were purchased from Symbiotics (USA) and Leonardo (Italy) respectively. AZT used was the pharmaceutical production of Sigma. All other reagents are of molecular biology grade. 24 well plates were from Corning (USA). For the administration of drugs 18 Standard Wire Gauge bend oral dosing/gavage needle was used. Syringe filters were purchased from Pall, HPLC (Waters) was used for the estimation of drug. All Safety analysis experimental kits were purchased from Span diagnostics India and Cayman chemical USA. p24 ELISA kit was purchased from ABL (USA).

### Animals

All the animals (Wistar rats) used in this study were approx. 6 months old and 0.160 kg to 0.250 kg weight, obtained from Sainath Agencies Hyderabad. Animals were housed in animal house facility of University of Hyderabad and all animal experiments were conducted as per the approval from Intuitional Animal Ethics Committee, University of Hyderabad.

### Preparation of Lactoferrin Nanoparticles

AZT loaded lactoferrin nanoparticles were prepared through sol-oil chemistry [31]. 10 milligram of AZT was dissolved in 1000μl of Milli-Q water and was gently mixed with 40 mg of lactoferrin dissolved in 1ml of ice cold phosphate buffer saline (pH 7.4). Mixture was incubated in ice for an hr. Then it was slowly added to 25ml of olive oil with gentle vortexing. The sample was sonicated for 15 min at 4°C with the help of narrow stepped titanium probe of ultrasonic homogenizer (300V/T, Biologics Inc., Manassas, Virginia, USA). Resulting mixture was immediately transferred in liquid nitrogen for 10 min then thawed on ice for 4hr. Particles formed were centrifuged at 6000 rpm for 10 min at 4°C. Pellet formed was extensively washed twice with ice cold diethyl ether (to completely remove oil) and then dispersed in 1ml of PBS.

### Nanoparticle Characterization

Nanoparticle morphology were examined through two different methods, field emission scanning electron microscope (FE-SEM, Philips FEI-XL 30 ESEM; FEI, Hillsboro, OR, USA) operated at 20 KV, and Atomic force microscope (AFM; SPM400). Gold coated Nanoparticles were used for FE-SEM and in AFM where samples were spin coated on glass slides. The characterization was done according to the protocol described as per manufacturer’s instructions.

### FT-IR Spectral Analysis (Drug-Nanoparticle Interaction Study)

Spectral analysis were done using KBr pellet method. All the nanoparticles were lyophilized before FT-IR analysis. 0.1 to 1.0% sample was well triturated into 200 to 250 mg of KBr in a mortar. Samples were then transferred to 13 mm-diameter pellet forming die and very high pressure (1000 kg/cm^2^) was applied under vacuum; resulted to a transparent pellet. The pellet was placed on sample holder and scanned from wave number 4000 to 400. Spectral analysis was performed using FT-IR dedicated OMNIC series software on a windows 7 platform.

### Encapsulation Efficiency

Encapsulation efficiency was measured by mixing 50μl of nanoparticles and 950μl of 1X PBS (pH5) and incubated for twelve hours on rocker. After incubation, 200μl of 30% Silver nitrate was added and vortexed. 1ml of methanol was added, mixed thoroughly and centrifuged at 16000rpm for 10min at 4°C. After centrifugation supernatant was collected and filtered through 0.2 micron syringe filters and then filtrate was used for estimation by HPLC (Waters) at 270 nm. Mobile phase composition used was acetonitrile: methanol (60:40 v/v) [36]. 10μl of sample was injected at the flow rate of 1ml per min. The encapsulation efficiency (EE %) was calculated using the following formula

$$\text{Encapsulation Efficiency }\left(\text{\%}\right)=\frac{{\text{M}}_{\text{total}}-{\text{M}}_{\text{lost}}}{{\text{M}}_{\text{total}}}\times 100$$

Here M_total_ is the total amount of AZT entrapped during AZT-lactonano preparation and M_lost_ is the amount of AZT unavailable after release from nanoparticles.

### pH Dependent Drug Release Assay and Percent Release of Drug

600μg of AZT-lactonano were incubated for 12h with 1ml of 1X PBS (pH 2 to 8), SGF (simulated gastric fluid) and SIF (simulated intestinal fluid); 200 μl of silver nitrate was added to precipitate the protein and drug was extracted by adding methanol, centrifuged and supernatant was estimated for AZT using HPLC. To measure the percent release of AZT from nanoparticles, AZT-lactonano were incubated with 1ml of PBS (pH 5.0 and 7.4). At different time interval aliquots were withdrawn, and estimated for the presence of AZT.

### *In Vitro* Stability Study

The *in vitro* stability testing was performed for drug loaded nanoparticles (AZT-lactonano) in PBS (pH 7.4) (NP solution). The stability study was carried out in terms of quantity of drug present in the nanoparticles using HPLC and diameter. Freshly prepared nanoparticles were incubated for various time points such as 0, 1, 2, 4, 6, 8, 10, 12, 16, 24, 48, 72 and 96h at two different temperature (4°C and room temperature). Drug content was quantified using the protocol mentioned in drug release assay section. The size of above incubated nanoparticles were measured using FE-SEM.

### Animal Study Design

Male and female Wistar rats of 6 months old were acclimatized for a week at animal house facility, University of Hyderabad under 12h light/dark cycle. For study, animals were divided into seven different groups according to seven time points i.e., 30min, 1h, 2h, 4h, 8h, 16h, 24h. Each group contains 3 males and 3 females and drug was administered orally to all animals. Rats were administered with 10mg/kg body weight of sol AZT and equivalent of AZT-lactonano. Each group was subjected for above mentioned time points. Animals were monitored hourly after oral administration of drugs. No death was observed during the experimentation. After completion of respective time points, animals were euthanized using sodium pentobarbital (50 mg/kg, IP) and blood was collected through heart puncture. Then, tissues such as brain, heart, esophagus, lungs, spleen, liver, stomach, small intestine, large intestine, kidney and bone marrow were collected. Serum was separated from blood by centrifugation at 4° C, 1500g for 10min. Except bone marrow, tissue from all other organs were homogenized; 30% of silver nitrate was added to precipitate the tissue protein. Extraction of AZT from tissue was done by addition of methanol followed by the centrifugation of the whole mixture at 12,000 rpm for 12 min at 4° C. After centrifugation, supernatant was filtered with 0.2 micron syringe filter and estimated by HPLC UV detector at 270nm.

### Tissue Sectioning and Safety Analysis

Organs were removed and processed for histopathology. The tissues were observed under microscope for any abnormalities after the treatment with the nanoformulation. Safety analysis was done by using biochemical kits that were commercially available for serum AST, urea, bilirubin and creatinine.

### Bone Marrow Micronucleus Assay

Animals were administered orally with 10mg kg^-1^ body weight of sol AZT and equivalent amount of AZT-lactonano. The test was performed using the modified protocol of Schmid [37]. Only three time points (4h, 8h, and 16h) were chosen for the test, based on drug distribution in bone marrow. After the completion of time points rats were sacrificed by cervical dislocation. Both femoral bone were dissected and the bone marrow cells were collected by flushing out with 1ml of fetal bovine serum (FBS) and mixed properly. Then cell suspension was centrifuged for 5min at 200g; supernatant were discarded. Marrow pellet was resuspended in minimal volume of FBS. One drop of cell suspension were placed on a clean dry slide and thin smear was made, air dried and then fixed with absolute methanol. Smear was stained with Giemsa stain for 20min and mounted.

### Antiviral Assay

SupT1 Cells (100% viability) with a density of 0.5 million were seeded with RPMI 1640, 0.1% FBS in 24-well plates. 80mg/ml of lactoferrin was taken, and formulation with and without AZT (1μg) were added to the cells and they were challenged with HIV-193IN101 at a final concentration of virus equivalent to 20 nanograms of p24 per ml. The infected cells were incubated at 37°C and 5% CO_2_ incubator for 2 h. After 2 h, the cells were pelleted at 350x g for 10 min, cells were washed twice with RPMI 1640 containing 10% fetal bovine serum. The cells were suspended in the same medium and incubated for 96 h. After 96 h supernatants had been collected and viral load was analyzed using p24 antigen capture assay kit (ABL kit). The infection in the absence of compound was considered to be 0% inhibition.

### Statistical Analysis

All studies were carried out in triplicates for all groups and results are presented as mean with standard deviation. The significance of differences between treatments was analyzed by one-way ANOVA with age and treatment as factors using Sigma plot. The level of statistical significance (P) was set at P < 0.05.

## Results

### Size and EE% of AZT-Lactonano

**Fig 1:**
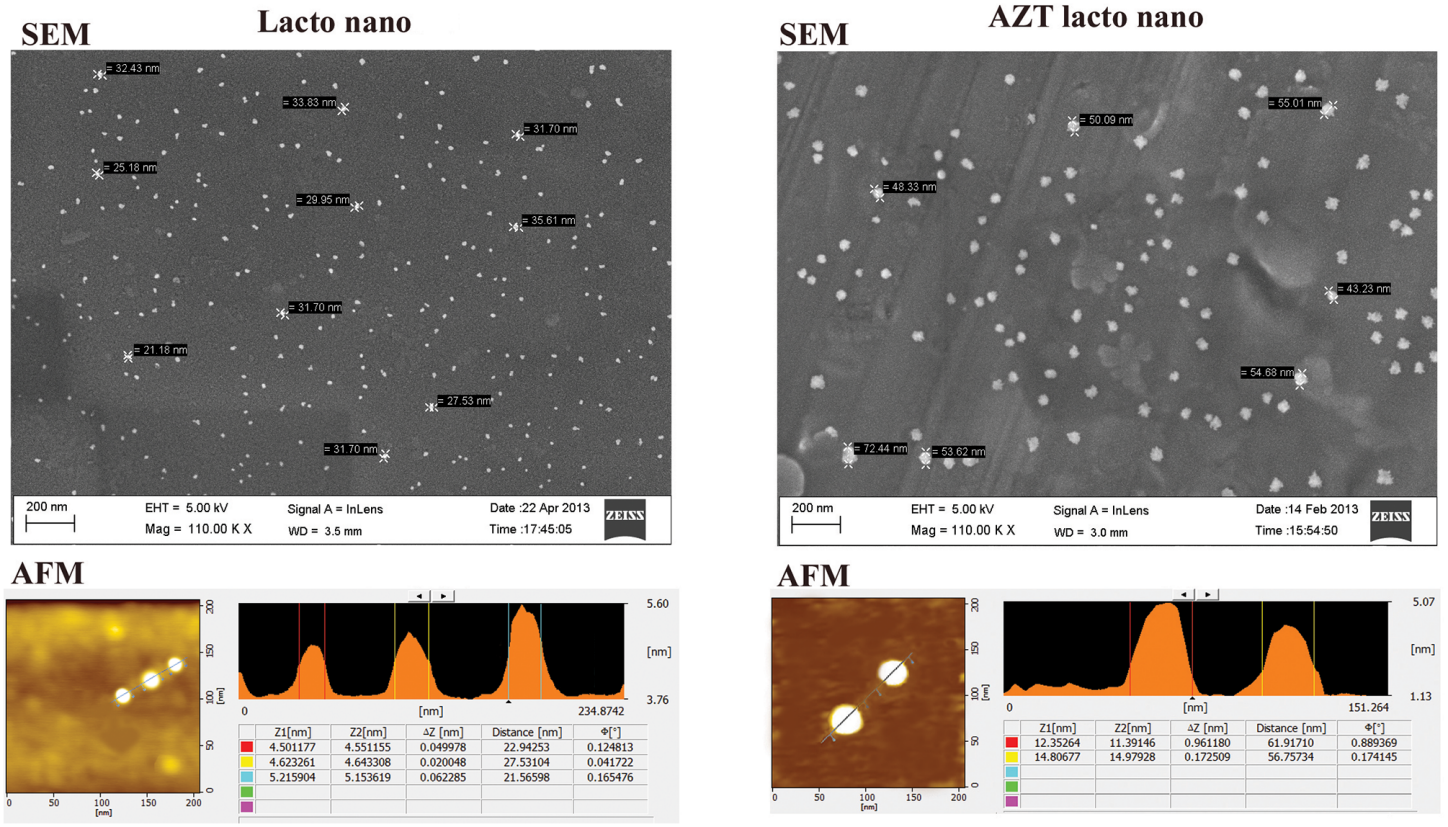
Size of Lactoferrin nanoparticles increases after loading of AZT. AZT containing Lactoferrin nanoparticles were prepared using sol-oil chemistry as described in methods section. The size of nanoparticles was assessed by Field emission scanning electron microscopy (top panel) and Atomic force microscopy (bottom panel). Lactonano: Lactoferrin nanoparticle without any drug loading into it, AZT Lacto nano: Lactoferrin nanoparticle loaded with AZT.

Field Emission Scanning Electron Microscopy (FE-SEM) and Atomic force microscopy (AFM) analysis revealed that the particles were spherical and were in the range of 50-60nm diameter (Fig 1). Increase in size of AZT-lactonano (50 to 60 nm) compared to lactonano (21-35nm) suggest that drug loading enhance particle size. Encapsulation efficiency was calculated according to the equation mentioned in materials and methods section and found to be 67%.

### FT-IR Analysis of AZT-Lactonano

**Fig 2:**
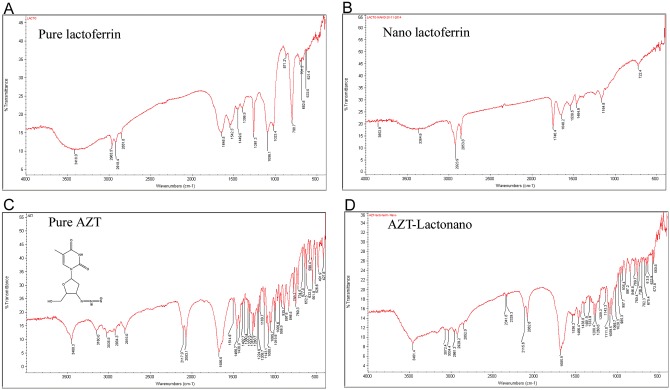
FT-IR spectral analysis. The FT-IR analysis of Pure Lactoferrin proteins (A), Nano Lactoferrin (Lacto nano) (B), AZT molecule (C) and AZT loaded lactoferrin nanoparticle (AZT-Lactonano) (D). It reveals that in AZT-Lactonano, AZT is physically entrapped/adsorbed with the lactoferrin nanoparticles; it didn’t take part in any sort covalent interaction.

FT-IR analysis confirmed that AZT was found to be intact after the preparation of nanoparticles (Fig 2). Characteristic bands found in the infrared spectra of Lactoferrin proteins (Pure and nano form) include the Amide I and Amide II. The absorption associated with the Amide I band and Amide II band leads to stretching vibrations of the C = O bond and primarily to bending vibrations of the N—H bond respectively. Amide I bands was positioned around 1645 & 1648 cm^–1^ are usually reflected to be characteristic of alpha helices. Amide II (C-N stretching and N-H bending) and peptide N—H stretching frequency were detected at 1542 & 1539 cm^–1^ and 3418 & 3364 cm^–1^ correspondingly. The C-O-C stretch were observed around 1096 cm^–1^ & 1164 cm^–1^ The locations of both the Amide I and Amide II bands are sensitive to the secondary structure content of a protein. **Assessment spectral analysis of Pure AZT and AZT-Lactonano**: sharp characteristic peaks of carbonyl group (C = O) at 1682 cm^–1^ (Pure AZT) and 1685 cm^–1^ (AZT-Lactonano), Azide group (N^−^ = N^+^ = N^−^) peaks at 2117 & 2083 cm^–1^ (Pure AZT) and 2115 & 2082 cm^–1^ (AZT-Lactonano), C-O-C stretch belong to 1088 & 1065 cm^–1^ (Pure AZT) and 1089 & 1065 cm^–1^ (AZT-Lactonano), -NH stretching remains at 3460 cm^–1^ (Pure AZT) and 3461 cm^–1^ (AZT Lactonano). Our results of FT-IR spectra proved that there were only slight shifting (may be due to dipolemoment of bond as a result of electrostatic interaction between AZT and Lactoferrin protein) in few stretching vibration but all the major functional group was intact in nanoformulation and didn’t take part in any covalent bond formation. It confirmed that AZT is only physically associated (entrapped/adsorbed) with lactoferrin protein.

### AZT-Lactonano Release Assay

**Fig 3:**
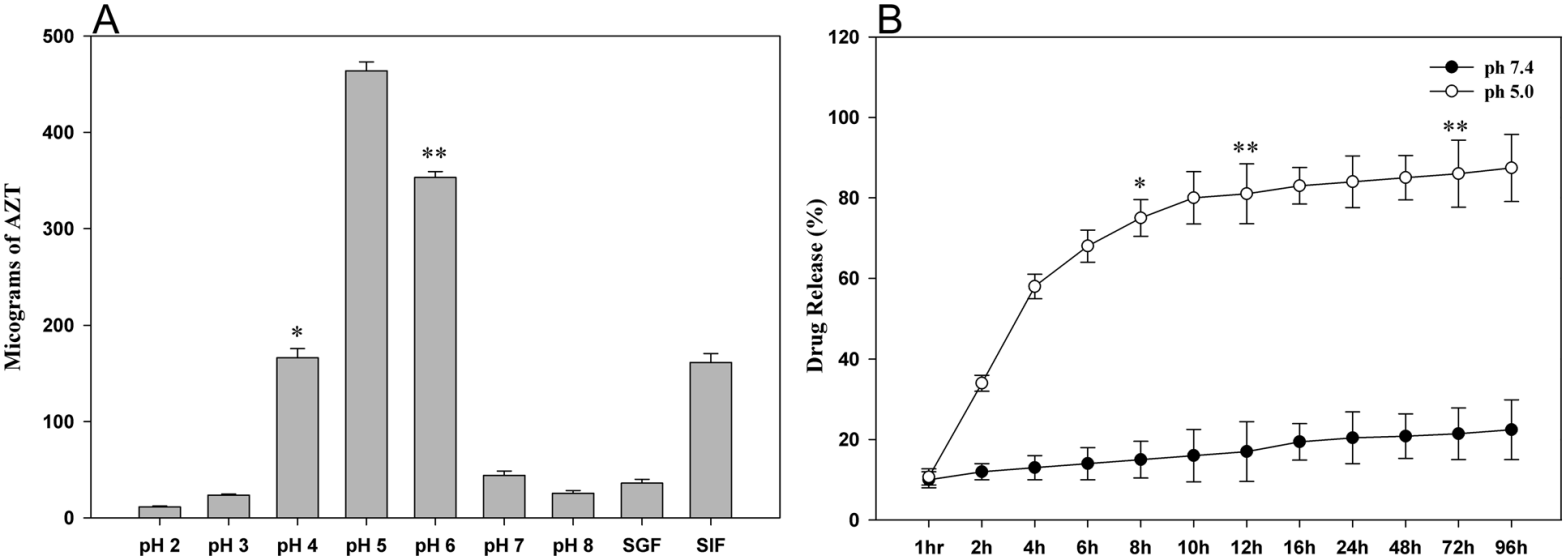
pH sensitivity and time dependent drug release profile of AZT-Lactonano. (A) 600μg of drug was incubated in the buffers of different pH, the release of AZT was maximum at pH-5. This is followed by pH-6 and pH-4. The release was between 1–10% with the remaining fluids. SGF: Simulated Gastric Fluid, SIF: Simulated Intestinal Fluid. (B) Cumulative percentage release profile of AZT-Lactonano at pH 5.0 and pH 7.4. Each data points were taken in triplicate and presented as Mean ± SD. Value of significance, **P < 0.005, *P < 0.05.

In case of percent drug release assay, the encapsulated drug present in nanoparticle was considered as 100% at the start of the experiment. The amount of drug released at indicated pH at different time points was estimated and presented as percent release with reference to the drug loaded in the nanoparticles. In vitro analysis of AZT release from the nanoparticles has shown that highest amount of AZT was released at pH-5 (Fig 3A) followed by pH-6 and pH-4. Its release in the presence of simulated Gastric and Intestinal fluids was lesser indicating its stability at extreme pH. Release kinetics at pH 5.0 as indicated in Fig 3B showed a biphasic release, wherein a burst release of 60% of drug within 4 hours, followed by reduced release rate until 10 hours to the extent of 80% then a limited release over 96 hours.

### *In Vitro* Stability Studies

Stability of nanoparticles suspended in PBS was analyzed at different time points at two different temperatures. Nanoparticles were found to be quite stable at both temperatures (4°C and room temperature). The drug content of nanoparticles and its diameter was found to remain same from starting to 96hr (Table 1).

**Table 1:** *In vitro* stability of nanoparticles.

	Diameter of the nanoparticles (nm)		AZT present in mg (%) ^#^	
Hours	4°C	Room temp*	4°C	Room temp*
0	55.67± 4.54	53.23± 3.56	6.75 ± 0.69 (100.00)	6.71 ± 0.19 (100.00)
1	54.5 ± 4.30	54.5 ± 2.68	6.49 ± 0.96 (96.15)	6.57 ± 0.82 (97.91)
2	58.6 ± 3.36	59.7 ± 5.13	6.68 ± 0.85 (98.96)	6.66 ± 0.28 (99.25)
4	53.1 ± 4.19	53.6 ± 4.35	6.71 ± 0.52 (99.41)	6.47 ± 0.18 (96.42)
6	52.3 ± 3.63	55.5 ± 4.72	6.58 ± 0.78 (97.48)	6.53 ± 0.36 (97.31)
8	54.7 ± 4.73	57.6 ± 3.81	6.37 ± 0.67 (94.37)	6.54 ± 0.49 (97.46)
10	58.3 ± 6.63	58.7 ± 4.34	6.66 ± 0.78 (98.66)	6.30 ± 0.84 (93.89)
12	53.6 ± 5.13	56.8 ± 3.92	6.48 ± 0.82 (96.00)	6.52 ± 0.92 (97.16)
16	60.2 ± 5.39	54.1 ± 4.77	6.52 ± 0.19 (96.59)	6.50 ± 0.14 (96.87)
24	58.7 ± 4.73	59.4 ± 4.65	6.64 ± 0.35 (98.37)	6.68 ± 0.49 (99.55)
48	55.6 ± 4.73	57.1 ± 2.91	6.51 ± 0.73 (96.44)	6.63 ± 0.19 (98.81)
72	56.7± 5.94	56.7 ± 2.65	6.54 ± 0.37 (96.88)	6.58± 0.27 (98.06)
96	60.8 ± 5.76	58.4 ± 4.39	6.65 ± 0.39 (98.52)	6.35 ± 0.38 (94.63)

All the data (n = 3) were presented as mean ± standard deviation.

^**#**^ Drug present in the particles was estimated by using HPLC, in milligrams. The amount of drug present initially at zero hour at 4°C and room temperature are considered as 100% drug present.

* Room temperature: The temperature used here was an average equal to 23°C.

### Pharmacokinetics and Tissue Distribution of AZT Lactonano

**Fig 4:**
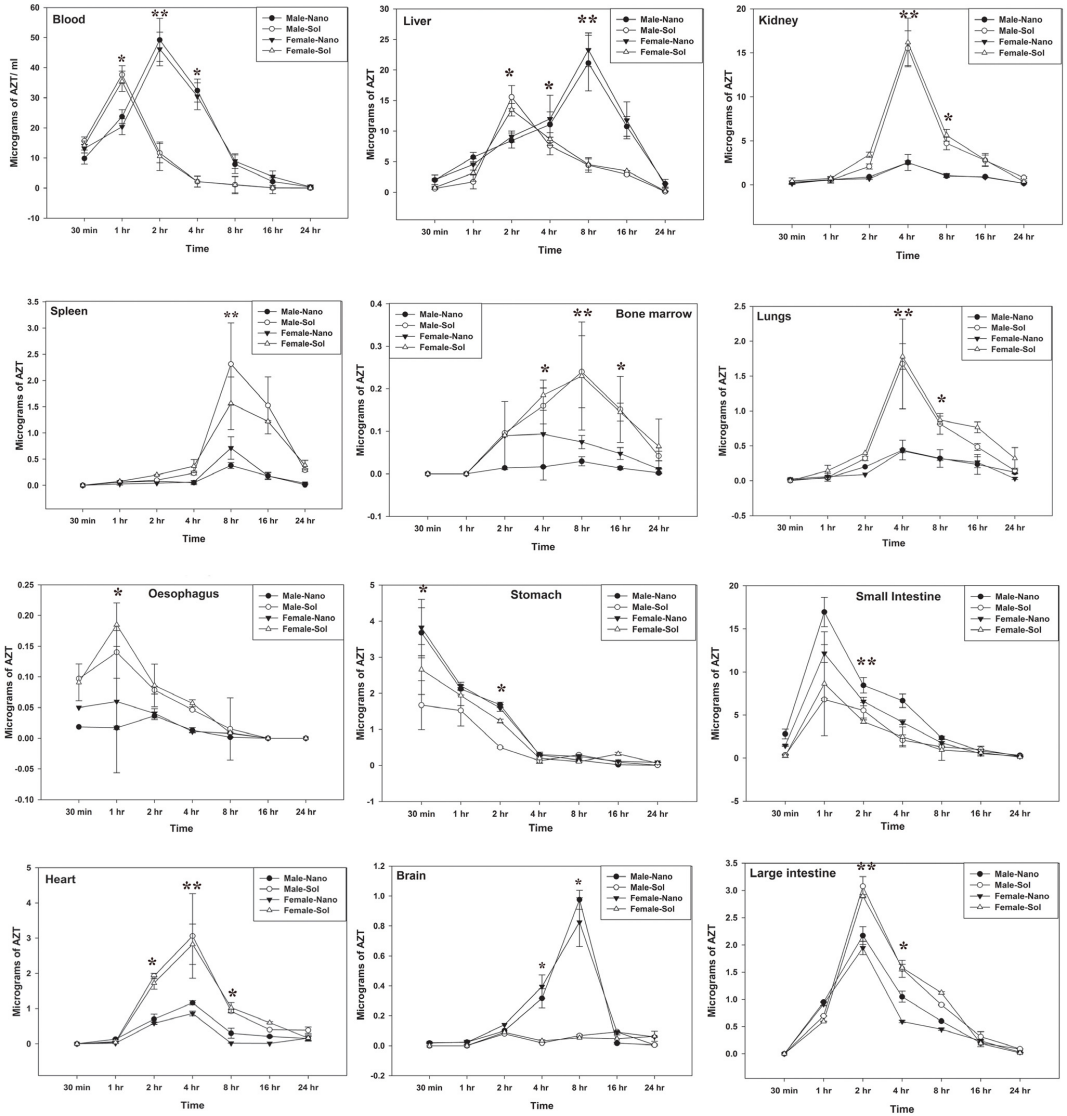
Tissue distribution of AZT. Single dose of sol AZT and equivalent weight of AZT-lactonano (10mg/kg body weight) was orally administered to Wistar rats. After completion of indicated time points, rats were sacrificed under proper anesthesia. AZT was extracted and estimated in blood, liver, kidney, heart, spleen, bone marrow, lungs, brain, oesophagus, stomach, small intestine and large intestine. Male-Nano and Female-Nano denotes the AZT concentration (delivered via AZT-lactonano) present in Male rats (n- = 3) and female rats (n = 3) respectively. Same nomenclature has been followed for Male-Sol and Female-Sol. Differences between groups were assessed by ANOVA. Data were presented as Mean ± SD. Value of significance, **P < 0.005, *P < 0.05.

AZT levels in blood and other organs were located after a single dose of nanoparticles loaded with AZT (10mg/kg AZT equivalence) administered in rats orally. Drug levels were observed at seven different time point up to 24h in different tissue (Fig 4). The concentration of AZT in the blood after 2 h was determined to be around 50μg when administered with nanoformulation and around 10μg in the lack of any carrier particle. It proves the relative stability of AZT in nanoformulation against plasma clearance. Nanoformulation does not cause any organs related toxicity due to the accumulation of AZT in various organs, in contrast AZT without any carrier molecule leads to toxicity. It is followed by liver and small intestine which have shown accumulation above 5μg. Less than 5μg of the drug was found in the others remaining organs such as Kidney, Heart, Spleen, Lungs, Brain, Stomach, Esophagus, large intestine and Bone marrow. On the other side we can detect a simultaneous decrease of AZT level in serum; in liver there was an increased level of AZT when treated with AZT-lactonano at 8hr post injection. All pharmacokinetic parameters were assessed by Kinetica v 5.0 software are shown in Table 2. The AZT-lactonano showed an improvement in pharmacokinetic profile with more than 4-fold increase in AUC and AUMC in male and female rats in serum. The serum C_max_ for AZT-lactonano was increased by 30%. Similarly, there was more than 2-fold increase in T_max_ and t_1/2_ (Table 2). These results show that the nanoformulation provide higher bioavailability than sol formulation.

**Table 2:** Pharmacokinetics profile of AZT-lactonano in male and female rats.

AZT		Male		Female	
		Nano	Soluble	Nano	Soluble
**AUC**	(h)*(μg/ml)	251.57	63.48	254.974	58.74
**AUMC**	(h)^2*(μg/ml)	1270.3	139.73	1485.24	126.737
**C**_**max**_	**μg/mL**	49.198	37.67	46.17	35.45
**T**_**max**_	**hr**	2	1	2	1
**t**_**1/2**_	**hr**	3.07	1.759	3.27	1.92

Values in the parenthesis designates the concentration of AZT in micrograms per ml of blood.

Pharmacokinetic parameters.

**AUC**: The integral of the concentration-time curve (after a single dose or in steady state).

**AUMC**: Partial area under the moment curve between t start and t end.

**C**_**max**_: The peak plasma concentration of a drug after oral administration.

**T**_**max**_: Time to reach C_max_.

**t**_**1/2**_: The time required for the concentration of the drug to reach half of its original value.

### Safety Profile of Nanoformulation

**Fig 5:**
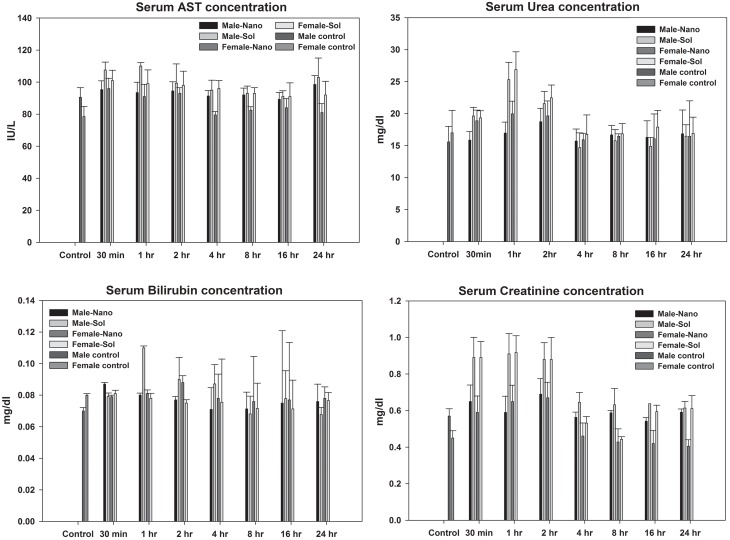
Biochemical safety analysis profile. Safety analysis was done using biochemical kits after oral administration of nano and soluble AZT (10mg/kg) in both male and female rats. Liver damage was estimated by Bilirubin and AST level whereas Kidney toxicity was checked by Urea and creatinine level. AZT-lactonano showed no toxicity to both liver and kidneys on the other hand it exhibited minimal urea levels when compared to the soluble AZT.

**Fig 6:**
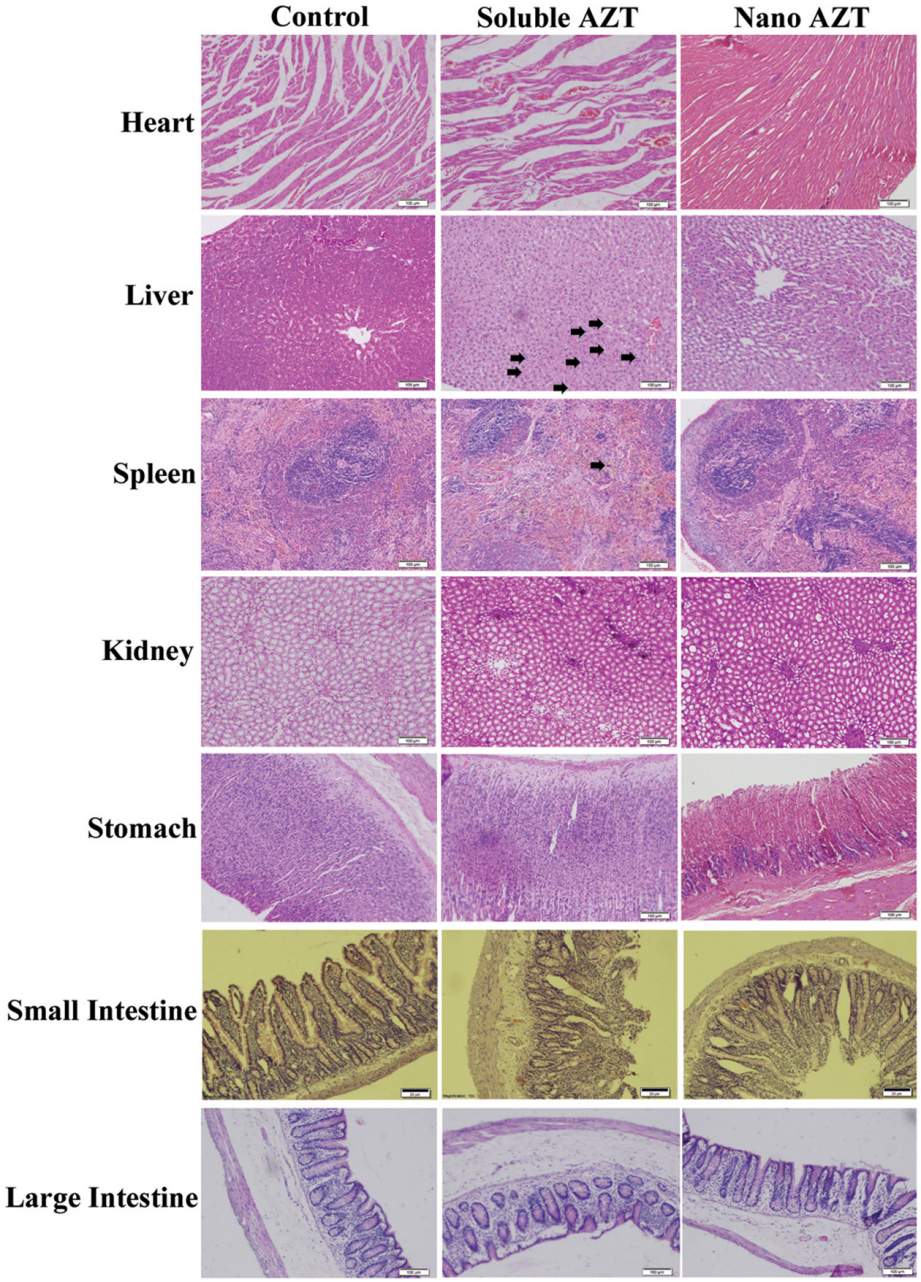
Histopathological analysis of tissues. Rats were orally administered with sol AZT and AZT-lactonano (10mg/kg body weight), after completion of 24hr time point, organs were removed and processed for cryo-sectioning followed by Hematoxylin and Eosin (H&E) staining. It revealed that no toxicity was found in above indicated organs, when AZT was delivered via nanoparticle form as compared to its sol form. Lesion or any abnormalities present was denoted by arrow. Scale bar is equal to 100μm.

The safety profile of AZT-lactonano was compared with sol AZT and the results show no significant change in serum AST in nanoformulation versus soluble form, while bilirubin was lower in case of AZT-lactonano compared to sol AZT in female rats (Fig 5). Serum urea was significantly low when AZT-lactonano was administered suggesting low kidney toxicity when nanoformulation was used. Nanoformulation showed improved creatinine levels compared to soluble drug form suggesting low liver toxicity. Tissue sections of heart, liver, spleen, kidney, stomach and intestines (small and large) have shown that there were no toxicities in those tissues when administered with nano forms (Fig 6).

### Micronucleus Assay

**Fig 7:**
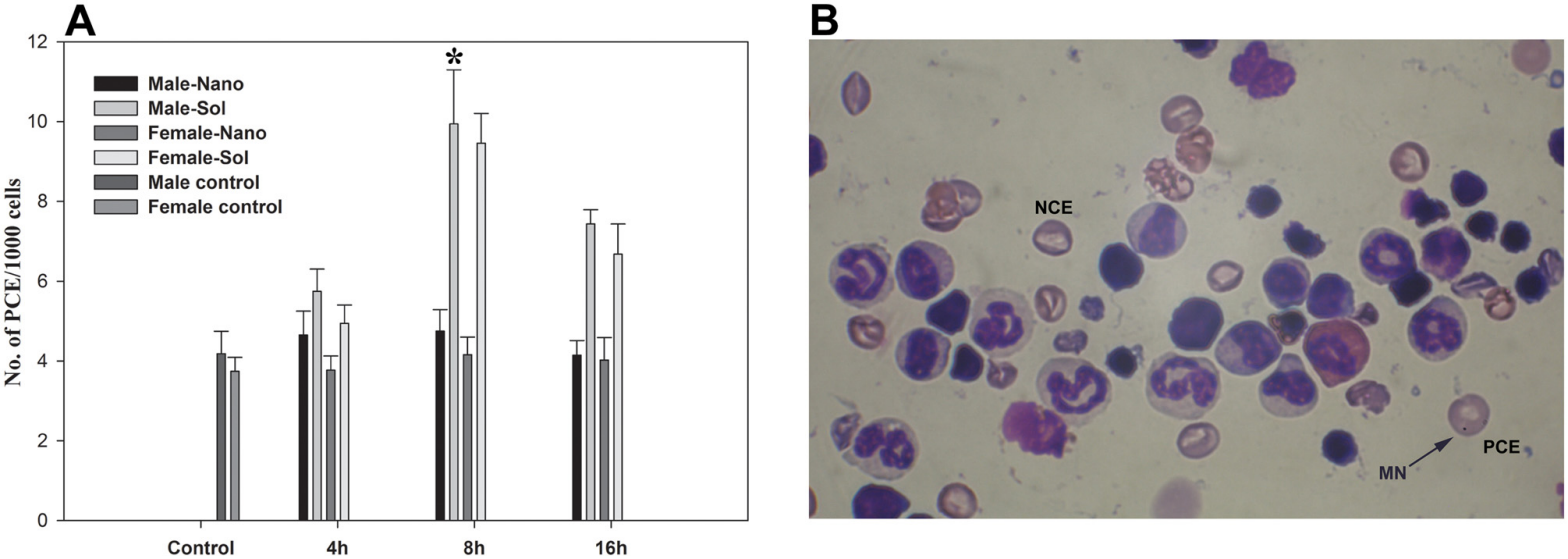
Bone marrow toxicity profile. **(A)** It shows the frequency of polychromatic erythrocyte (PCE) in bone marrow cells after oral administration of sol AZT and AZT-lactonano at 4, 8 and 16h. Data were presented as Mean ± SD. Value of significance, **P < 0.005, *P < 0.05. **(B)** Bone marrow cells (after 8h of treatment) showing the presence of enucleated Normochromatic erythrocyte (NCE) and nucleated polychromatic erythrocyte (PCE) cells. One PCE holds a micronucleus (MN); indicated by arrow. This images was captured at 100x under oil immersion objective.

Bone marrow smears were visualized using Olympus Magnus BX51 microscope under 100x magnification with oil immersion objective. Micronuclei (MN) were identified in form of RBCs (i.e., polychromatic erythrocytes as PCEs). Approximately 1000 cells were scanned per slide for the presence of micronucleus (MN) in immature PCE. Fig 7A represent that, at 8h of post application of drug a significant increase in the number of MN-PCEs was observed in case of sol AZT and at the same time AZT-lactonano showed low bone marrow toxicity. MN could be characterized as round and darkly stained (Fig 7B) nuclear fragment that indicate chromosome damage.

### Antiviral Activity of AZT Lactonano

Antiviral activity of lactoferrin alone and AZT loaded in nanoformulation was analyzed to ascertain if active form of the drug is intact. Here 80mg/ml of soluble lactoferrin and equivalent of nanoform of lactoferrin has showed 70 and 73% antiviral activity respectively. Further, the activity of AZT at one microgram concentration is similar to that of soluble AZT with more than 90% of inhibition of HIV-193IN101 replication in Sup-T1 cells. It suggests that activity of encapsulated AZT remain stable in nanoformulation. (S1 Fig).

## Discussion

Various formulations have been employed previously to improve the oral bioavailability of AZT. These include controlled [38] and extended [39,40] release matrices, microspheres [41], nanoparticles [42] and liposomes [43–45], which have been proposed for the delivery of AZT. Even though various routes like intranasal, intravenous and transdermal routes have been tried for AZT delivery. Peroral route of administration is the most preferred one because of frequent dosage and patient compliance. Absorption of AZT is reported to be quick and rapid when administered orally and undergoes first pass metabolism before giving an average systemic bioavailability of more than 60% [46]. Based on physiochemical characteristics, the aqueous solubility, pKa and LogP of AZT was reported as 29.3 g/l, 9.68 and 0.06 respectively [47]. Zidovudine typically exhibits a 1-compartment model in plasma during its oral administration followed by an elimination phase that is biexponential. It is relatively a lipophilic molecule where 25% of it binds to albumin [48,49] and gets metabolized in the body mainly by hepatic 5’-glucouronidation forming a stable metabolite which gets excreted in the urine. In order to maintain the required therapeutic concentration of AZT, frequent doses have to be given which may lead to elevation to toxic levels in the blood resulting in severe side effects like granulocytopenia and anemia. Greater focus has been given for targeting AZT to lymphoid and reticuloendothelial cells in previous formulations since the delivery to these cells is utmost important as they constitute major viral reservoirs and a sterilizing cure for AIDS is impossible unless and until these are eliminated completely.

Since the encapsulation mechanism involves a process of partition of lipophilic-lyophilic AZT and lactoferrin in water and oil phases. Such phase transitions may induce protein-protein associations that entrap drug in intermolecular core as well as intramolecular cavities of proteins. This lead to a defined percent of drug (67%) associated with the protein nanoparticles based on the log P value of the drug, viz., AZT (log P, 0.06). This can be supported by the observation that AZT is released in biphasic kinetics with burst (60%) when protein surface of particles exposed to pH 5, which could be due to change in orientation of protein monomers, this may follow a release of drug molecules localized at various cavities in the protein over time (to the extent of 20%). In spite of biphasic release, 80% of loaded drug was released within 10 hours, making the availability of active drug for inhibiting target enzyme, revise transcriptase activities. Furthermore, oral absorption of nanoparticles at low pH (<3.0) and circulatory pH 7.4, allow particles intact with the loaded drug. When these particles reached the target cells (lymphocytes etc.), they enter through receptor-mediated endocytosis followed by fusion to endosome, a transient pH change to 5.5 in endosome will allow significant drug release in target cell and make effective concentrations of drug reaching at the site of action. Thus, stability of particles at pH below 3.0 and at 7.4 make these particles attractive for oral delivery in vivo.

*In vivo* studies showed that the AZT-lactonano showed has improved pharmacokinetic profile with more than 4-fold increase in mean serum AUC and AUMC in both male and female rats. The serum C_max_ for AZT-lactonano was increased by 30% whereas more than 2-fold increase was observed in T_max_ and t_1/2_ for both male and females. It suggests that the AZT in the nanoparticles gets released slowly leading to this significant increase in the pharmacokinetic parameters. At the same time, this nanoformulation has not shown any abnormal concentrations in different organs leading to toxicity. The safety profile of nano and Sol AZT was compared and the results show no significant change in serum AST in nano versus soluble form, while bilirubin was lower in case of nano when compared to soluble form in female rats. Serum urea was significantly low when AZT-lactonano was administered compared to soluble AZT suggesting low kidney toxicity when nanoformulation was used. AZT-lactonano showed no apparent differences in creatinine levels compared to soluble form suggesting low kidney toxicity. In addition H&E staining of all the tissue sections has not revealed any abnormal morphology for both of the formulations employed.

Bone marrow suppression is the main reason to discontinue AZT based therapy [50] because the hematopoietic progenitor cells are heavily damaged. Micronucleus (MN) test is very reliable and fast in vivo assay to determine any marrow cells alteration [51]. MN are minute extra-nuclear bodies formed during anaphase stage [52]. Generally two forms of MN are found in RBCs, polychromatic erythrocytes (PCE) and Normochromatic Erythrocytes (NCEs) [53]. Our results show that AZT-lactonano is not involved in any MN formation but at the same time Sol-AZT is two-time more genotoxic.

As the free drug is reported to have lower penetration into the infected cells, the above formulation selectively targets and delivers AZT to cells that express lactoferrin receptors on their surface through receptor-mediated endocytosis by which the therapeutic index of AZT can be improved. The amphiphilic nature of AZT results in low entrapment and significant leakage when packed in conventional liposomal vesicles as it gets partitioned between lipid bilayers and the core aqueous environment. The lesser size of the nanoparticles with up to 67% encapsulation of AZT makes the current formulation to overcome the above problem. Currently, oral dosed HIV nanoformulation was not available for patients. The quality of patient’s life can be improved by simplifying the AZT dosage schedule by less frequent administration of a sustained-release formulation since HAART regimens that combine multiple agents lead to severe side effects. The advantage in employing lactoferrin as a carrier is its ability to interfere with virus binding to DC-SIGN of dendritic cells by its interaction with the V3 loop of gp120 and coreceptors [54]. Further, the same formulation can be employed to improve the brain delivery of AZT since the lactoferrin is reported to cross the blood-brain barrier [55].

## Conclusion

The Present study shows the applicability of protein -based nanoparticles formulation of AZT through oral delivery. The nanoparticles were prepared using sol-oil protocol. AZT-lactonano showed a biphasic drug release profile and releases its maximum payload at pH 5. *In vivo* studies concludes that the physical encapsulation of AZT in lactoferrin nanoparticles makes the formulation safer and efficacious. Nano-formulation enhances the various pharmacokinetics profile like AUC, AUMC, C_max_ and t_1/2_ while keeping the antiviral activity of AZT intact. Further AZT-lactonano is found to be two times less genotoxic as compared to sol AZT.

## Supporting Information

S1 Fig Antiviral activity of lactoferrin alone (soluble & nano) and AZT (soluble & nano). Antiviral activity of AZT was found to be intact in case of nanoformulation. The p24 level was measured as viral load. Here 80mg/ml of lactoferrin and equivalent concentration of nanoform was taken. The nano-AZT showed more than 85% antiviral activity at a final concentration of 1μg.
(TIF)
