# Supplementary figures and images for: Improved Safety, Bioavailability and Pharmacokinetics of Zidovudine through Lactoferrin Nanoparticles during Oral Administration in Rats
Source: PLoS One. 2015 Oct 13;10(10):e0140399. doi: 10.1371/journal.pone.0140399 (PMC4604150; doi:10.1371/journal.pone.0140399)

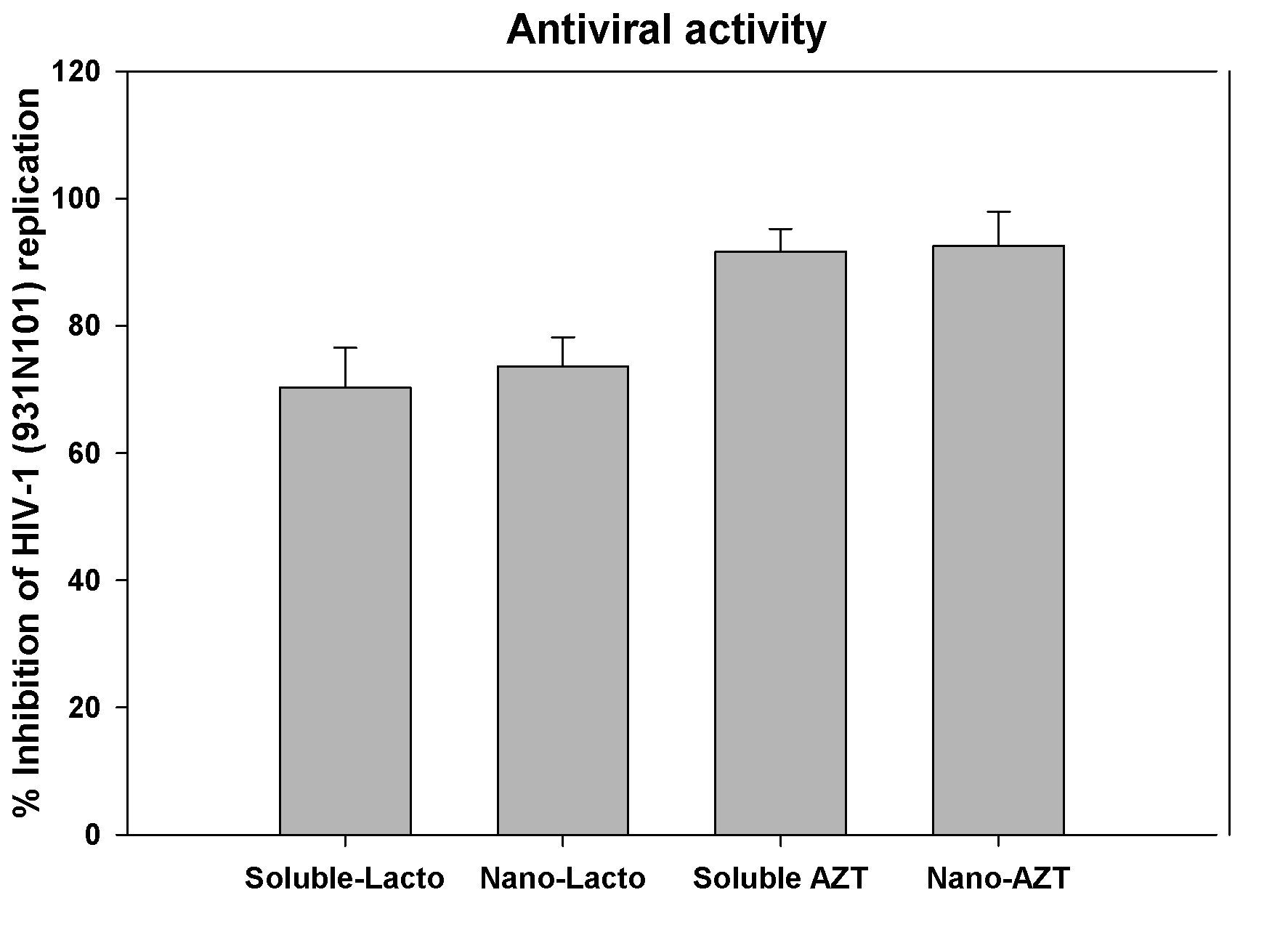

Supplement: S1 Fig — Antiviral activity of AZT was found to be intact in case of nanoformulation. The p24 level was measured as viral load. Here 80mg/ml of lactoferrin and equivalent concentration of nanoform was taken. The nano-AZT showed more than 85% antiviral activity at a final concentration of 1μg. (TIF) [file pone.0140399.s001.TIF]
